# Eddy Current Sensor System for Tilting Independent In-Process Measurement of Magnetic Anisotropy

**DOI:** 10.3390/s21082652

**Published:** 2021-04-09

**Authors:** Frank Wendler, Rohan Munjal, Muhammad Waqas, Robert Laue, Sebastian Härtel, Birgit Awiszus, Olfa Kanoun

**Affiliations:** 1 Professorship of Measurement and Sensor Technology, Chemnitz University of Technology, 09111 Chemnitz, Germany; frank.wendler@etit.tu-chemnitz.de (F.W.); rohan.munjal@etit.tu-chemnitz.de (R.M.); muhammad.waqas@etit.tu-chemnitz.de (M.W.); 2 Professorship of Virtual Production Engineering, Chemnitz University of Technology, 09111 Chemnitz, Germany; robert.laue@mb.tu-chemnitz.de (R.L.); sebastian.haertel@mb.tu-chemnitz.de (S.H.); birgit.awiszus@mb.tu-chemnitz.de (B.A.)

**Keywords:** eddy current sensors, magnetic sensor, magnetic anisotropy, tilting correction, inductance spectroscopy, impedance spectroscopy

## Abstract

Modern production equipment is based on the results of quality control as well as process parameters. The magnetic anisotropy of materials is closely connected to internal mechanical stress by the Villari effect, and also to hardening effects due to plastic deformations, and could therefore provide an interesting basis for process control. Nevertheless, the analysis of anisotropic properties is extremely sensitive to sensor and workpiece misalignments, such as tilting. In this work, a novel eddy current sensor system is introduced, performing a non-contact measurement of the magnetic anisotropy of a workpiece and realizing a separation and correction of tilting effects. The measurement principle is demonstrated with the example of two samples with different magnetic anisotropy values induced by cold forming. Both samples are analyzed under different tilt angles between the sensor axis and the surface of the workpiece. In this work, digital signal processing is demonstrated on the acquired raw data in order to differentiate the effects of tilt and of anisotropy, with the use of preliminary results as an example of two prepared samples.

## 1. Introduction

The fourth industrial revolution has driven changes in several sectors by the introduction of novel sensor and communication technologies [1,2]. Thereby, the progress in the field of manufacturing requires the automation of the production process by the measurement of material properties in real-time.

The extracted information from the process enables the assessment of the material and its internal state, as well as the minimizing of defects arising due to tilting [3] of a material.

In the case of metals, the process accompanying the recording of mechanical material’s internal states, such as residual stress or hardening, is of high importance for process control [4]. These internal material stresses and distributions are only partially accessible via observations on the surface, and often require the destructive preparation of a sample, which records only geometric changes or surface deformations, indirectly implying the internal material properties. This in turn requires a direct, non-contact, and in-line analysis of the properties of the metallic materials, which can be performed by measuring their magnetic anisotropy. This phenomenon varies depending on the direction of measurement [5], and exhibits intrinsic easy and hard directions of magnetization [6].

The quantitative analysis of magnetic anisotropy allows for the retroactive analysis of the primary influencing factor, which is the residual stress in the material section [3] of ferromagnetic materials, such as alloys of iron, nickel and cobalt.

Thereby, the quantitative analysis of anisotropy was insufficiently explored to enable an estimation of the underlying material properties and mechanical states.

Magnetic anisotropy can be deduced from either the static response or the dynamic response of the magnetic system. The majority of the experimental data so far have been obtained from static measurements [6]. The static methods most frequently used to determine magnetic anisotropy include magnetization and torque measurements [6]. Chikazumi et al. [7,8] studied magnetic anisotropy via the cold rolling of ferromagnetic material. In this study, anisotropy is measured using a torque magnetometer, in which a ferromagnetic specimen is placed in a strong magnetic field, magnetizing the specimen to saturation. Torque is then measured as a function of the angle of rotation of the magnetic field about the vertical axis, thus obtaining the torque curve, from which anisotropy constants are deduced. The limitation of this static method is that it is a destructive, contact-based measurement process which requires a complicated sample preparation process involving the cutting-out of the metallic specimen, thus making it unsuitable for in-line process measurement.

There exist other works that focus on non-contact anisotropy measurement, and are therefore more suitable for in-line process measurement. The dynamic response of the magnetic layers can be measured by using eddy current sensors [9,10], magnetic sensors [11], and ferromagnetic resonance (FMR) [6]. Doirat et al. [9] observed the magnetic response of anisotropic metal fiber material by using a ferrite core eddy current sensor together with an impedance analyzer. In the field of carbon fiber materials, eddy current measuring systems consisting of transmitter and receiver coils are used for laboratory investigations that evaluate the impedance of the directional coupled coil pair [12,13]. These systems require the re-orientation between the specimen and the sensor to detect different directions and do not allow for the correction of incorrect positioning.

For purposes of industrial production, the sensors need to be able to correct misalignments and tilting. One solution to this problem was implemented by Du et al. [10], who proposed a non-contact method based on eddy currents together with photo-electric sensors, whereby eddy current sensors are used for metal characterization and photo-electric sensors are used for tilting correction.

All of the above proposed sensor systems use single- or dual-sensor coil systems, which can separate and quantify the conductivity and permeability of materials by measuring conditions such as the distance between the test specimen and the measuring system [14]. However, these systems assume material homogeneity, and require significant improvements in the detection and quantification of anisotropy, since the direction-dependent material properties require a greater variety of sensor elements.

To overcome this problem, we propose a novel, non-contact, non-destructive, multi-channel eddy current sensor system with several magnetic sensors around a central sensor coil, allowing for the quantitative analysis of anisotropy as well as compensating for the tilting effect between the metal sample and sensor system that arises from the production process of a material. The concept of coupling inductive sensors is extended by the use of more pick-up sensor elements, in a specific pattern, so that the raw data can be used to differentiate the effects of anisotropy and those of tilting.

## 2. Principle of the Novel Sensor

In anisotropic materials, the properties vary depending upon the direction of measurement [5]. Some materials exhibit electrical and magnetic anisotropy due to the directional properties of electrical conduction and their ability to magnetize. In ferromagnetic materials, magnetic anisotropy arises due to the crystal orientation and mechanical stress in the material [6]. For instance, internal stress in a sample can result in magnetic anisotropy as a result of the Villari effect. Materials may exhibit a strong axis with high magnetic capability, or a weak axis with less capability to magnetize. The presence of internal stress in a material is also related to the plastic deformation of the material [3,15].

**Figure 1:**
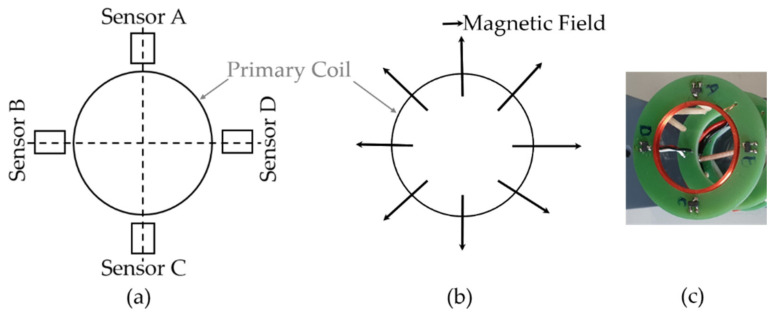
(**a**) Four magnetic sensors placed around the excitation sensor coil each 90° apart to detect the magnetic field in these directions. (**b**) Magnetic field of a coil directed radially outwards. (**c**) Realization of sensor system.

To measure anisotropy, a sensor system must be capable of distinguishing between the direction-dependent properties of the material. In this paper, the sensor system consists of a primary coil of 1 mH, placed in the center, and four DRV5056-Q1 Hall magnetic field sensors with a sensitivity of 200 mV/mT around the primary coil to measure its time-varying magnetic field (Figure 1a). A single frequency voltage signal is used to excite the primary coil and the current and voltage in this coil are measured to obtain an impedance spectroscopy-based measurement. The system is able to measure the properties of the material not only at its surface, but also at its core, by varying the excitation frequency. As shown in Figure 1b, the magnetic field of the primary coil is directed radially outwards, so that the field reaching the sensor passes the tested material in a specific direction and, thus, becomes dependent on material properties in the corresponding direction.

**Figure 2:**
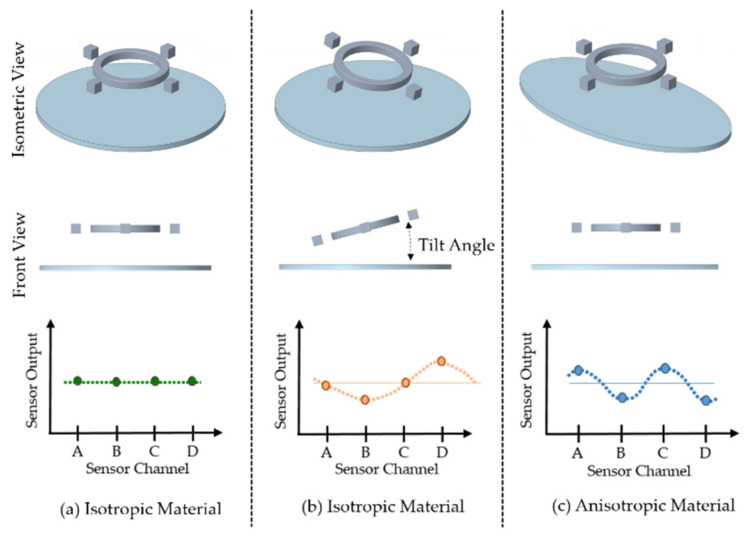
Representation of the four sensor outputs for three different scenarios: (**a**) Isotropic material aligned parallel to the sensor; (**b**) Isotropic material aligned tilted to the sensor; (**c**) Anisotropic material aligned parallel to the sensor.

By detecting the magnetic field of the primary coil at various points on the circumference via use of the additional sensor elements, its direction-dependent amplitude and phase position can be determined, allowing for the quantification of both the anisotropic and isotropic parts of the magnetic properties of the material with a single measurement. This effect is shown by the example given in Figure 2, which shows three distinguishable cases depending on sensor inclination and anisotropy.

In Figure 2a, the sensor is oriented parallel to the isotropic sample surface. In this case, the measured magnetic field is the same at all four sensor positions, and its magnitude depends only on distance and magnetic permeability.

In Figure 2b, the sensor is slightly tilted against the isotropic sample surface. In this case, one part of the sensor is closer to the sample, and sensors on the other side (180° along the circumference) are further away from the sample. Sensor D is closer to the sample and provides a stronger signal, while sensor B on the opposite side of the device provides a weaker signal due to its greater distance from the sample. This case is characterized by the formation of a sinusoidal waveform with exactly one full period over the entire circumference of the sensor.

In Figure 2c, the sensor is oriented parallel to the anisotropic sample’s surface. In this case, two oppositely oriented sensors show a strong signal because they lie on the magnetically strong axis. The other two sensors lie on the magnetically weak axis of the workpiece, and therefore show a weaker signal. This case is characterized by the formation of a sinusoidal curve with exactly two full periods over the entire circumference of the sensor. Anisotropy can be theoretically determined by using only one or two magnetic sensors, provided those sensors are aligned perfectly. The inclusion of two additional magnetic sensors allows for the correction of the tilting behavior by use of a Fast Fourier Transform (FFT) approach on the measured data. The use of four magnetic sensors allows for analysis of the tilting effect, as two obverse-directed sensors will display different behaviors dependent on the greater or smaller distance between the sensor and the surface. In contrast, in the case of anisotropic behavior, both sensors would display the same signal amplitude, since they share one magnetic axis.

## 3. Experimental Set-Up

**Figure 3:**
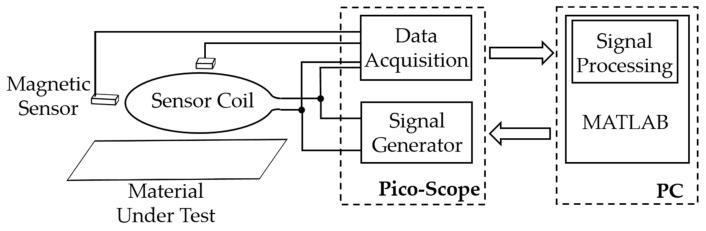
Block diagram of a sensor system.

Our measurement system consisted of a multi-channel measuring system with one primary coil and four magnetic sensors including the required analog components, such as an operational amplifier for signal conditioning. The Picoscope 5444B with in-built signal generator and Picoscope 4824 with 8 measurement channels were installed in the setup and controlled using MATLAB over a PC. The signal generator was used to excite the primary coil with a signal frequency of 500 Hz and a constant amplitude of 2 V, which generates a magnetic field around the coil. The four magnetic sensors were coupled with the 8-channel oscilloscope to measure the magnetic field generated by the primary coil in the presence of the material being tested, while the individual sensor output in the time-domain was recorded by the 8-channel oscilloscope using MATLAB as shown in Figure 3. The current response of a primary coil over a resistor of 1.5 Ω was also recorded by the 5th channel of the 8-channel oscilloscope.

The amplitudes of the magnetic sensors were extracted using FFT on the recorded time-domain sensor outputs. By determining the amplitude and phase of the sine wave using Equations (1)–(3) [16] with the corresponding wave number, information about tilt and anisotropy could be obtained.

(1) $$X_{w}\left(k\right)\;=\overset{N-1}{\underset{n\;=\;0}{\sum }}x\left(n\right).[\cos\left(2.\pi .k.\frac{n}{N}\right)-\;j.\sin\left(2.\pi .k.\frac{n}{N}\right)]$$

(2) $$X_{w}\left(k\right)\mathrm{magnitude}\;=\;{\left({\left(X_{w}\left(k\right)\mathrm{real}\right)}^{2}+\;{\left(X_{w}\left(k\right)\mathrm{img}\right)}^{2}\right)}^{\frac{1}{2}}$$

(3) $$X_{w}\left(k\right)ø\;=\;\tan^{-1}(\frac{X_{w}\left(k\right)\mathrm{img}}{X_{w}\left(k\right)\mathrm{real}})$$

The parameter “k” denotes how many periods are within the range of “N” samples.

k = 0 -> D.C. value

k = 1 -> Tilting

k = 2 -> Anisotropy

Here, the amplitude of the sine wave along the circumference indicates the amount of tilt or anisotropy. The phase position provides information about the direction of tilt or anisotropy. The use of a Fourier analysis allows for the determination of both the amplitude and phase of the respective wave number, and enables their evaluation even if combined states of tilt and anisotropy are present.

**Figure 4:**
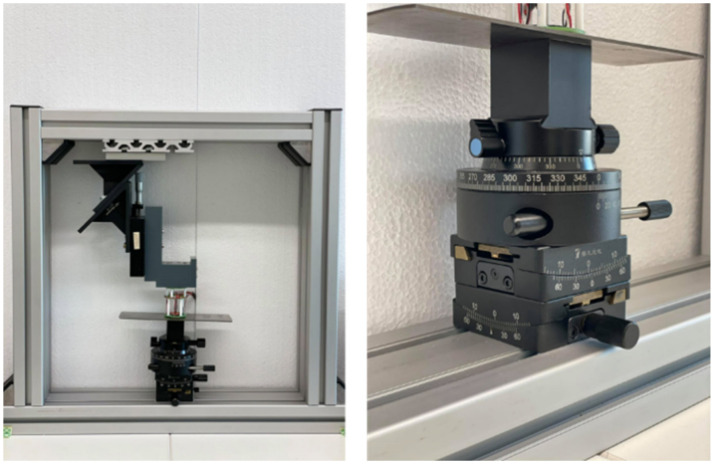
Experimental setup with a rotating table, two tilting tables, and slider to adjust the material being tested.

An essential novelty of this approach is that, due to the large number of sensor channels, several quantities—such as electrical and magnetic properties, anisotropy, and tilting—can be simultaneously detected, separated, and evaluated by the sensor system in one measurement. The experimental setup included a mechanical system for measuring the rotations and the tilting of the workpiece. The mechanical set-up was constructed using an aluminum frame consisting of two tilting tables and one rotating table stacked together on top of each other. The material to be tested was connected to the rotating table as shown in Figure 4. The rotating table can rotate the metal sample in 360° so as to observe the anisotropic behavior in different directions with high spatial resolution. The tilting tables were used to provide tilt to the material along two perpendicular axes. One tilting table was used to level the sample, while the other was used to provide tilt to the sample and further help in investigating the effect of tilt through the magnetic response of the magnetic sensors. The sensor system was placed over a specifically designed polyoxymethylene part which has no influence on the magnetic response of the material being tested. The distance between the sensor system and the material being tested can also be adjusted using a slider.

## 4. Experimental Results

**Figure 5:**
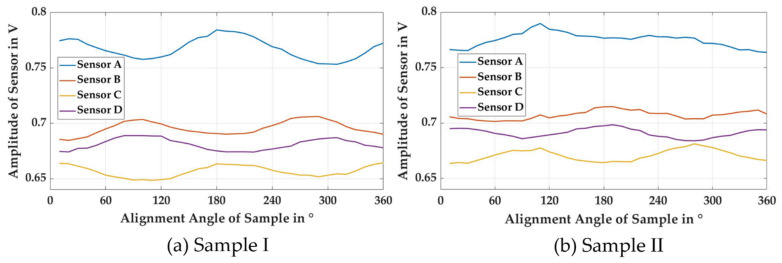
Rotational measurement of sample I and sample II in intervals of 10° to observe the effect of anisotropy for the cases of (**a**) strong anisotropy and (**b**) weak anisotropy.

The steel DC-01 was used for preparing sample I and sample II with different levels of plastic strain. Sample I was weakly deformed with a plastic strain of 0.15, while sample II was strongly deformed with a plastic strain of 0.60. In an initial analysis of anisotropy by rotating the samples, sample I revealed a higher anisotropy than sample II. To visualize the anisotropy, both samples were placed parallel to the sensor (tilt angle 0°) and were rotated 360° at 10° intervals. The amplitude of the sensor output was plotted against the rotating angle (Figure 5), showing that sample I exhibited the expected sine wave pattern, with two maxima at higher amplitude due to the presence of high anisotropy, while sample II exhibited a similar pattern but with lower amplitude.

**Figure 6:**
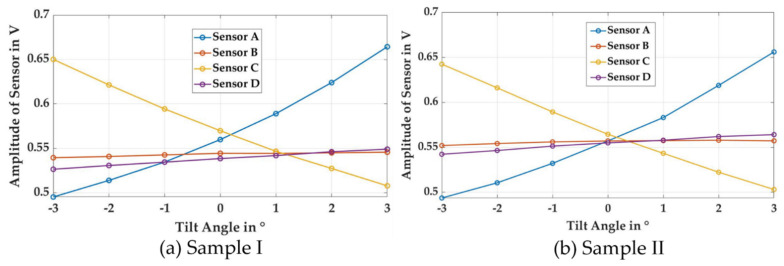
Effect of tilting on sensor readings for the cases of (**a**) strong anisotropy and (**b**) weak anisotropy.

To investigate the effect of tilting in Figure 6, the sensor was tilted from an angle of −3° to +3° at a rotating angle of 0°. Sensor B and sensor D were aligned with the tilting axis while sensor A and sensor C lay in the tilting direction and were affected by individual changes in distance due to tilting of the sample. As the angle of the tilt increased, sensor A drew closer to the material’s surface, while sensor C drew further away from it. This resulted in an increase in the amplitude of sensor A while it approached the surface, and a corresponding decrease in the amplitude of sensor C as its distance from the surface increased. Since sensors B and D were aligned with the tilting axis, their amplitude ought to have remained constant, as there ought to have been no change in their distance from the surface. However, due to a slight misalignment of the tilting axis of the sample and the central axis of the sensor, a slight change in distance with a tilted angle was observed. This resulted in a slight change in signal amplitude for those sensors with increased in tilting.

The effect of anisotropy was immediately visible. In the case of sample II, all sensors exhibited a similar amplitude value at a tilting angle of 0°, while in the case of Sample I the amplitudes of sensors A and C had higher values at the same tilting angle of 0°. This was due to the presence of higher anisotropy in Sample I and the alignment of its strong magnetic axis with sensors A and C.

**Figure 7:**
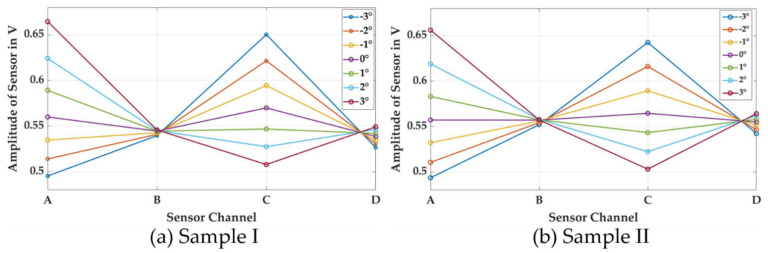
Effect of tilting on sensor readings in regards to the individual sensor channels for the cases of (**a**) strong anisotropy and (**b**) weak anisotropy.

**Figure 8:**
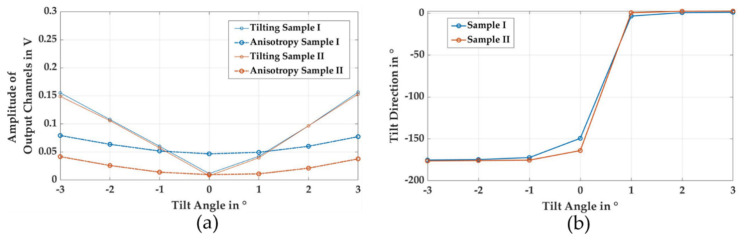
Output of the sensor systems after second Fast Fourier Transform (FFT): (**a**) Tilting and anisotropy for sample I and sample II as functions of the tilting angle. (**b**) Tilting direction as a function of the tilting angle for sample I and sample II.

In Figure 7, the data from Figure 6 are plotted in regards to the sensor channels. In the case of Sample I, a distinct pattern with two minima and two maxima can be seen for a tilting angle of 0°. This corresponds to the pattern observed in Figure 5. In the case of sample II, all four channels have very similar amplitudes at a tilting angle of 0°. For other tilting angles, a distinct pattern of tilting—with one minimum and one maximum—dominates the data set. The four data points for each channel in Figure 7 were used as the input for the second FFT analysis, resulting in an output vector of three values. The first output value, with k = 0, represents the mean level of signal of all four sensors, as well as general coupling conditions between the primary coil and the individual sensors—such as the mean distance of the target and the distance between the sensor and the primary coil. The second output, with k = 1, is a complex value and is used to analyze a sine wave with one period over the circumference of the sensor, and therefore corresponds to the tilting value of the sample. From the complex nature of this number, the tilting direction can be derived—as displayed in Figure 8b—from the relation of the imaginary and real components using Equation (3). The third output, where k = 2, analyzes the amplitude of the sine wave with two periods over the circumference of the sensor, and enables us to measure the anisotropy of the sample.

In Figure 8a, the magnitudes of the second and third outputs of the second FFT are plotted as sensor tilting and anisotropy. In this figure, the tilt magnitude is linearly related to the absolute tilting angle, and is very similar for both samples. The magnitude of the anisotropy signal is much higher for sample I than for sample II, which corresponds to the characteristic amplitudes obtained by rotating the sample in Figure 5. The anisotropy signal is not constant, as is desired in the measurement task. When the magnitude of the tilt was increased, anisotropy increased in a systematic way, due to the non-linearity of the distance behavior of the sensors. In Figure 6, it can be seen that an increase in distance results in a smaller change in sensor amplitude than a decrease in distance of the same length. The increase in signal amplitude overcompensates for the decrease in amplitude, leading to a higher output after the second FFT. This systematic deviation is a function of mean signal amplitude and tilting. Those two values can be obtained by the sensor, and may be used for further corrections of the anisotropy signal.

In Figure 8b, the direction of tilting is plotted. The zero point of the tilt direction is located at the position of sensor A. When changing the tilting angle from −3° to +3°, the sample was first tilted towards sensor C and then tilted towards sensor A. This was observed as a change from a tilt direction of approximately −180° to a tilting direction of approximately 0°. Deviations in the tilting direction at a 0° tilt angle were caused by slight deviations in the initial alignment of the axes of sensors and samples. 

## 5. Conclusions

In this paper, a novel method is proposed for the independent measurement of the magnetic anisotropy of metallic materials on tilting angles. This method uses an impedance-based eddy current sensor system with a central primary coil together with four magnetic sensors.

For proof of principle, two samples with different levels of magnetic anisotropy were prepared by cold forming, and were subjected to measurement at different tilt angles. The obtained data were compared at different steps of the digital signal processing, where the first FFT was performed on the acquired raw data, while the second FFT was applied to the output of the first FFT, in order to differentiate the effects of tilt and anisotropy in two prepared samples. The preliminary results show that magnetic anisotropy was superimposed by tilt dependence, which exceeded 300% of the signal level of the anisotropy itself during these experiments. By making use of the characteristic pattern of both effects, and the second FFT, both signals could be separated and analyzed quantitatively. The obtained results show that the difference in magnitude of anisotropy for both samples remains constant over the investigated tilting angles. In addition, the obtained information about the magnitude of tilt in the direction of tilting shows a very good correspondence to the underlying geometric conditions. The correction of tilt angles with an absolute value of less than 3° greatly improves the capacity of the sensor system for in-process measurements. The designed system does not require a re-orientation of the specimen to perform an anisotropy measurement, and requires only the initial alignment of the sensor with strong and weak magnetic axes. The problem of initial alignment can be overcome by the use of more than four sensors, which will make it feasible to simultaneously detect the direction of anisotropy and measure the anisotropy at arbitrary angles. Similarly, this method can be used for the analysis of electrical anisotropy in carbon fiber. By combining the already obtained information of anisotropy and tilting magnitude, active correction with the use of actuators and the repositioning of the sensor is possible.
